# CBX7 regulates stem cell-like properties of gastric cancer cells via p16 and AKT-NF-κB-miR-21 pathways

**DOI:** 10.1186/s13045-018-0562-z

**Published:** 2018-02-08

**Authors:** Su-Jie Ni, Li-Qin Zhao, Xiao-Feng Wang, Zhen-Hua Wu, Rui-Xi Hua, Chun-Hua Wan, Jie-Yun Zhang, Xiao-Wei Zhang, Ming-Zhu Huang, Lu Gan, Hua-Lin Sun, Goberdhan P. Dimri, Wei-Jian Guo

**Affiliations:** 1 Department of Medical Oncology, Fudan University Shanghai Cancer Center, 270 Dong-An Road, Shanghai, 200032 China; 2 Department of Oncology, Shanghai Medical College, Fudan University, Shanghai, 200032 China; 3 Department of Biochemistry and Molecular Biology, The George Washington University Medical Center, Washington, DC, 20037 USA; 4 Department of Medical Oncology, Affiliated Hospital of Nantong University, Nantong, 226001 China; 5 Department of Nutrition and Food Hygiene, School of Public Health, Nantong University, Nantong, 226001 China; 6 Jiangsu Key Laboratory of Neuroregeneration, Nantong University, Nantong, 226001 China

**Keywords:** CBX7, p16, AKT, miR-21, Gastric cancer, Stem cells

## Abstract

**Background:**

Chromobox protein homolog 7 (CBX7), a member of the polycomb group (PcG) family of proteins, is involved in the regulation of cell proliferation and cancer progression. PcG family members, such as BMI, Mel-18, and EZH2, are integral constituents of the polycomb repressive complexes (PRCs) and have been known to regulate cancer stem cell (CSC) phenotype. However, the role of other PRCs’ constituents such as CBX7 in the regulation of CSC phenotype remains largely elusive. This study was to investigate the role of CBX7 in regulating stem cell-like properties of gastric cancer and the underlying mechanisms.

**Methods:**

Firstly, the role of CBX7 in regulating stem cell-like properties of gastric cancer was investigated using sphere formation, Western blot, and xenograft tumor assays. Next, RNA interference and ectopic CBX7 expression were employed to determine the impact of CBX7 on the expression of CSC marker proteins and CSC characteristics. The expression of CBX7, its downstream targets, and stem cell markers were analyzed in gastric stem cell spheres, common cancer cells, and gastric cancer tissues. Finally, the pathways by which CBX7 regulates stem cell-like properties of gastric cancer were explored.

**Results:**

We found that CBX7, a constituent of the polycomb repressive complex 1 (PRC1), plays an important role in maintaining stem cell-like characteristics of gastric cancer cells via the activation of AKT pathway and the downregulation of p16. Spearman rank correlation analysis showed positive correlations among the expression of CBX7 and phospho-AKT (pAKT), stem cell markers OCT-4, and CD133 in gastric cancer tissues. In addition, CBX7 was found to upregulate microRNA-21 (miR-21) via the activation of AKT-NF-κB pathway, and miR-21 contributes to CBX7-mediated CSC characteristics.

**Conclusions:**

CBX7 positively regulates stem cell-like characteristics of gastric cancer cells by inhibiting p16 and activating AKT-NF-κB-miR-21 pathway.

**Electronic supplementary material:**

The online version of this article (10.1186/s13045-018-0562-z) contains supplementary material, which is available to authorized users.

## Background

Gastric cancer (GC) is one of the most common malignant diseases worldwide [1]. Although the worldwide morbidity and mortality due to gastric cancer declined in recent years, GC is still ranked as the second and third most common cancer in male and female population, respectively [1]. Therefore, early diagnosis and effective treatment of gastric cancer remain the major research focuses of oncologists around the world. It is widely believed that a minority of tumor cells endowed with self-renewal and tumor-initiating properties, known as cancer stem cells (CSCs), play an important role in cancer development [2–4]. Recent evidence suggests that CSCs are also key to tumor invasion, metastasis, disease recurrence, and resistance to chemotherapy [5, 6]. In case of gastric cancer, the existence and pathological significance of gastric cancer stem cells (GCSCs) were firstly suggested by Takaishi et al. [7]. Subsequently, further studies have confirmed the existence of GCSCs or stem-like cells in gastric cancer cell lines and gastric cancer tissues [8–11]. However, the molecular mechanism underlying GCSC phenotype remains unclear.

Polycomb group (PcG) proteins, which regulate the expression of homeotic genes (HOX) via gene silencing, have been reportedly deregulated in an array of cancers [12–16]. PcG proteins function at different sites in chromosomes by forming multi-protein complexes, known as polycomb repressive complexes (PRCs), resulting in the alteration of chromosomal structure and transcriptional repression of gene expression via epigenetic mechanisms [12]. Several of these canonical and non-canonical PRCs have been documented to play important roles in embryonic development, cell proliferation and differentiation, malignant transformation, and maintaining properties of normal stem and cancer stem cells [14–17]. Chromobox homolog 7 (CBX7), located on chromosome 22q13.1, encodes a chromobox protein and has been identified as a core PRC1 constituent [18, 19]. As a member of PcG family proteins, CBX7 can function independently in the initiation and progression of various cancer types [19–23]. However, the role of CBX7 in cancer development remains controversial. CBX7 may act as an oncogene or a tumor suppressor, depending on the cellular context and cancer types [19–23]. While CBX7 knockout mice were reported to develop lung and liver tumors, we and others have clearly shown that CBX7 has oncogenic properties [23]. In particular, CBX7 is overexpressed in gastric tumors. With respect to the oncogenic role of the PcG proteins, it has been reported that BMI1, EZH2, and Mel-18 were involved in the regulation of stem cells or CSC properties [15–17, 24–26]. Notably, CBX7 has been also documented to positively regulate stem cell characteristics of prostate cells and hematopoietic stem cells [27, 28]. Our previous study found that CBX7 is overexpressed and plays an oncogenic role in gastric cancer [23]. Therefore, we speculated that CBX7 might also be involved in the regulation of stem cell properties of gastric cancer cells. In the current study, we found that CBX7 positively regulated GCSC phenotype. Further in-depth studies showed that CBX7 regulated GCSC phenotype via the downregulation of its known target p16 and the upregulation of microRNA-21 (miR-21) by virtue of the activation of AKT-NF-κB pathway.

## Methods

### Cellular reagents and methods

Five strains of human gastric cancer cell lines (MKN28, MKN45, SGC-7901, AGS, NCI-N87) were obtained from the Surgical Institution of Ruijin Hospital, and a human gastric cancer cell line HGC-27 was obtained from the Chinese Academy of Sciences, Shanghai Cell Institution. MNK28, MKN45, SGC-7901, and NCI-N87 cell lines were cultured in RPMI-1640, AGS cell line was cultured in F12, and HGC-27 cell line was cultured in DMEM. The culture mediums were supplemented with 10% fetal bovine serum (FBS) and antibiotics. Cell proliferation was assessed by Cell Counting Kit-8(CCK8) assay. For plate colony formation assay, cells were planted into six-well plates (500 cells/well) and cultured for 2 weeks. Colonies were washed with PBS for three times, then fixed with 4% paraformaldehyde for 10 min. These fixed colonies were stained with 0.1% crystal violet solution and counted. Finally, the crystal violet was washed away with 10% acetic acid, and 600-nm absorbance was assessed by a Microplate Reader (BIO-TEK Instruments Minneapolis, MN). Cell migration and invasion assays were performed using Transwell chamber as described [29–31].

### Plasmids, retroviruses, and infection

Retroviral vectors (pGPU/Hygro and pEX-2/Hygro) expressing CBX7, CBX7 shRNA, and p16 shRNA were obtained from Shanghai GenePharma Co Ltd. The target sequence of CBX7 and p16 shRNA are shown as follows: CBX7, 5′-AGA ACC AGA GAG GCT CTG A-3′; p16 5′-GCC CAA CGC ACC GAA TAG TTA-3′.Other retroviral vectors including pSRα-mAkt (originated from Dr. N. Hay, UIC, Chicago, IL) and methods to produce retroviruses have been described before [32]. Stable cell lines overexpressing CBX7 or other genes of interest were generated by infection of respective retroviruses or transfection of the plasmids. Successfully infected cells were sorted by flow cytometry and then cultured in medium supplemented with G418 or other antibiotics.

### Clinical samples

Ninety-five paraffin-embedded primary site specimens of gastric cancer were obtained from the archives of the Fudan University Shanghai Cancer Center for immunohistochemical (IHC) analysis. The clinicopathological variables and survival data were obtained from medical records, and the disease stages were determined according to the 2010 UICC/AJCC gastric cancer TNM staging system. The study was approved by the Institutional Medical Ethics Committee of the Fudan University Shanghai Cancer Center. All clinical samples were collected with the informed consent of patients, and study protocols were in accordance with the ethical guidelines of the Declaration of Helsinki (1975).

### Spheroid colony formation assay

Spheroid colony formation (SCF, Tumorisphere) assay was carried out as described previously [11]. Briefly, cells were trypsinized and washed with PBS and then cultured in ultra-low-attachment plates containing serum-free medium DMEM/F12 supplemented with B27(1:50), N2(1:100), EGF (10 ng/ml), and bFGF (20 ng/ml). The medium was replaced every 5 days. Two weeks later, the number of spheres was counted and processed for protein extraction.

### In vivo tumorigenesis

Gastric cancer cells were injected subcutaneously into the flanks or abdominal cavity of severe combined immunodeficiency (SCID) mice. After 10–12 weeks, mice were sacrificed by cervical dislocation. For the flank-injected mice, tumors were removed, and their weight and size were measured. All experiments concerning animals were approved by the Animal Care and Use Committee of the Fudan University.

### Immunological reagents, Western blot, and immunohistochemical analysis

Antibodies against CBX7, Oct4, p16, and β-actin were purchased from Abcam. Antibodies against CD24 and CD44 were obtained from Abgent. Antibodies against pAkt (S473) and Akt were purchased from Cell Signaling Technology. The whole cell lysates were harvested using cell lysis buffer supplemented with protease inhibitor cocktail (Sigma), and Western blotting analysis was performed as described [32]. Immunohistochemical (IHC) analysis to detect the expression of CBX7 or other markers was performed as described [11, 23]. All slides were observed by two independent pathologists in a double-blinded fashion. The immunoreactive scoring method (IRS) was adopted to assess the result of immunohistochemistry. IRS value of more than 6 was regarded as high-expression group, while IRS value of less than 6 was regarded as low-expression group.

### Chemo-sensitivity experiment

Cells were plated into 96-well plates (5000 cells/well) in triplicates and fed with PRMI-1640 medium containing 10% FBS, along with different concentrations of the chemotherapy reagent (Adriamycin (ADM), 5-fluorouracil (5-Fu), and no drug/solvent as control). The number of viable cells was assessed after 2 days of cultivation using the Cell Counting Kit-8 (CCK8) (Dojindo, Kamimashiki-gun Kumamoto, Japan), and the optical absorbance at wavelength 450 nm was detected using the Microplate Reader (BIO-TEK Instruments, Minneapolis, MN).

### Statistics

All statistical analysis were done using SPSS 17.0 software package, and two-tailed *P* values of less than 0.05 were considered significant. In the set of IHC assay of paraffin-embedded tissue samples, the Pearson *χ*^2^ test was used to examine the correlation between CBX7 expression and clinicopathological parameters in primary gastric cancer, and paired *t* test was used to determine the difference of CBX7 expression between primary gastric cancer specimens and their corresponding paracancerous tissue. In in vitro experiments, data were described as mean ± SD and analyzed by Student’s *t* test.

## Results

### CBX7 positively regulates stem cell-like characteristics of gastric cancer (GC) cells

**Fig. 1:**
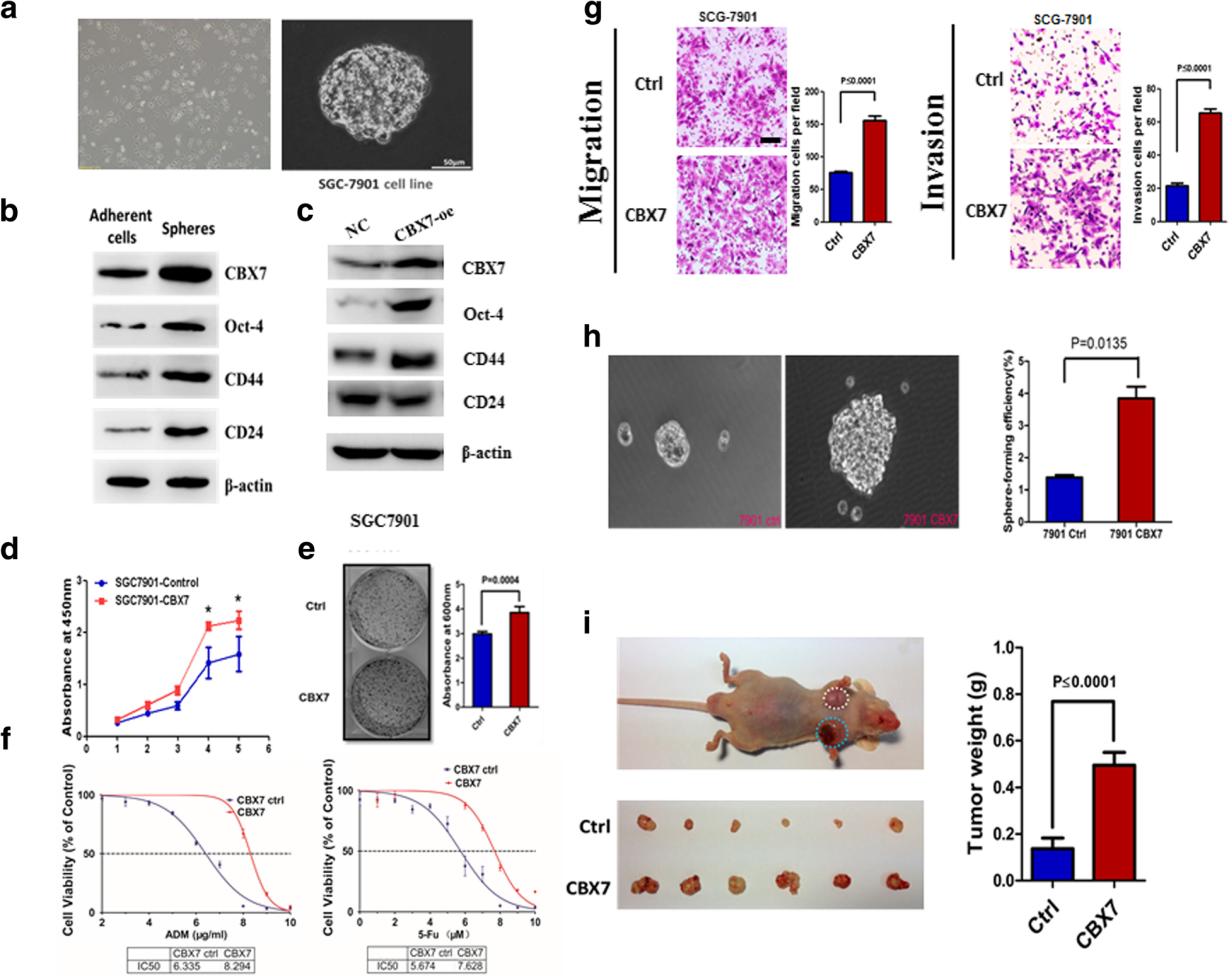
CBX7 positively regulates stem cell-like properties of gastric cancer cells. **a** Tumorigenic spheres (aka GCSC spheres) derived from SGC7901 gastric cancer cell line were cultured in serum-free DMEM/F12 medium supplemented with N2, B27, EGF, and bFGF. **b** Tumorigenic spheres overexpress stem cell markers including CBX7, OCT-4, CD44, and CD24. The expression of stem cell markers in the total cell lysate was analyzed by Western blot analysis. **c** The levels of CBX7 and stem cell markers OCT-4, CD44, and CD24 were detected in SGC-7901 cells expressing control vector (NC) or exogenous CBX7 (CBX7-oe) using Western blot analysis. β-actin was used as an internal control. **d** Cell proliferation of control and CBX7-overexpressing cell lines was determined using CCK-8 assay. **e** Colony-forming capability of CBX7 overexpressing and control cells was determined by seeding 1000 cells in sphere-initiating medium and an incubation period of 10 days. The colonies were fixed and stained with crystal violet. **f** Compared with control cells, CBX7-overexpressing SGC-7901 cells showed enhanced resistance to Adriamycin (ADM) and 5-fluorouracil (5-Fu). Cells were treated with ADM and 5-Fu at the indicated concentrations, and the numbers of viable cells were counted after 72 h of the treatment. The percentage of viable cells is shown relative to the untreated controls. **g** The migration and invasion of CBX7-overexpressing and control cells were determined using Corning Chambers. **h** Representative images of GCSC spheres originated from control and CBX7-overexpressing SGC-7901 cells. **i** CBX7 overexpression enhances in vivo tumorigenicity of SGC7901 cells. In vivo tumorigenic capacity of control (Ctrl) and CBX7-overexpressing SGC7901 cells was examined using SCID mice xenograft tumor model. Error bars in all panels represent the mean ± SD. *P < 0.05, compared with the control group

Previously, we reported that GC cell lines and primary GC cells could form oncospheres in serum-free culture medium and that the cells grown as spheres exhibited stem cell-like or CSC properties (Fig. 1a) [11]. To clarify the role of CBX7 in the stemness of GC cells, we first analyzed the expression of CBX7 and stem cell markers CD44, CD24, and OCT-4 in spheres and adherent cells. Our results showed that CBX7 was abundantly expressed in sphere cells from GC cell lines (Fig. 1b). Next, we transiently transfected CBX7 into SGC-7901 cells and determined whether ectopic expression of CBX7 could upregulate stem cell markers, such as CD44, CD24, and OCT-4. As predicted, our data showed that CBX7 overexpression resulted in the upregulation of OCT-4 and CD44 (Fig. 1c). We also analyzed the influence of CBX7 on the CSC properties of GC cells through sphere formation and chemoresistance assays. Our results showed that CBX7 overexpression resulted in increased proliferation and colony formation (Fig. 1d, e), decreased chemosensitivity (Fig. 1f), and augmented migratory and invasive capacities (Fig. 1g). CBX7 overexpression also resulted in increased sphere formation (size and number) (Fig. 1h). CBX7-overexpressing cells formed significantly larger tumors compared to control cells (Fig. 1i). Similar results were obtained using MKN-28 cells (Additional file 1: Figure S1). Consistent with these results, depletion of CBX7 resulted in impaired colony formation, sphere formation, and cell migratory and invasive capacities in GC cell lines (Additional file 2: Figure S2 and Additional file 3: Figure S3). To further determine the involvement of CBX7 on GC stem cell-like characteristics, we analyzed the expression of CBX7 in 95 GC tissues by immunohistochemical (IHC) assay. The results showed that CBX7 was highly expressed in 42.1% (40/95) of the GC tumors and that there was a trend of positive correlation between CBX7 expression and regional lymph node metastasis, as well as the TNM stage (Table 1). We also analyzed the expression of stem cell markers OCT-4, SOX-2, Gli1, CD44, and CD133 in GC tissue specimens and found that CBX7 expression positively correlated with the expression of stem cell markers OCT-4 and CD133 (Table 2). Collectively, these data suggest that CBX7 positively regulates gastric cancer stem cell (GCSC) phenotype.

**Table 1:** Correlation between CBX7 expression in gastric cancer tissues and clinicopathological variables

	CBX7(+)	CBX7(−)	*P* value
Lymph node metastases			
Negative	5 (23.8%)	16 (76.2%)	0.054
Positive	35 (47.3%)	39 (52.7%)	
Depth of invasion			
T1–3	16 (42.1%)	22 (57.9%)	1.000
T4	24 (42.1%)	33 (57.9%)	
TNM staging			
Stage I	0 (0%)	4 (100%)	0.072
Stage II	7 (31.8%)	15 (68.2%)	
Stage III	26 (47.3%)	29 (52.7%)	
Stage IV	7 (50.0%)	7 (50.0%)	
Degree of differentiation			
Moderately/moderately-poorly differentiated	16 (38.1%)	26 (61.9%)	0.481
Poorly differentiated	24 (45.3%)	29 (54.7%)	
Pathological types			
Adenocarcinoma	27 (40.3%)	40 (59.7%)	0.581
Signet-ring cell carcinoma/myxoadenocarcinoma	13 (46.4%)	15 (53.6%)	
Lymphatic or vascular invasion			
Positive	28 (46.7%)	32 (53.3%)	0.238
Negative	12 (34.3%)	23 (65.7%)	
Nerve invasion			
Positive	26 (45.6%)	31 (54.4%)	0.396
Negative	14 (36.8%)	24 (63.2%)	
Age(years)			
≥ 60	16 (42.1%)	22 (57.9%)	1.000
< 60	24 (42.1%)	33 (57.9%)	
Gender			
Male	24 (35.8%)	43 (64.2%)	0.055
Female	16 (57.1%)	12 (42.9%)

**Table 2:** Correlation between CBX7 expression and stem cell-related factors in gastric cancer tissues by Spearman rank correlation analysis

		Oct4	Sox2	Gli1	CD44	CD133	p-AKT	p-ERK
CBX7	*R*	.162	− .144	− .050	.082	.201	.188	− .066
	*P*	.033*	.058	.513	.284	.008*	.013*	.386

*P < 0.05 was considered to be statistically significant

### CBX7 regulates the cancer stem cell-like characteristics of gastric cancer cells via downregulation of p16

**Fig. 2:**
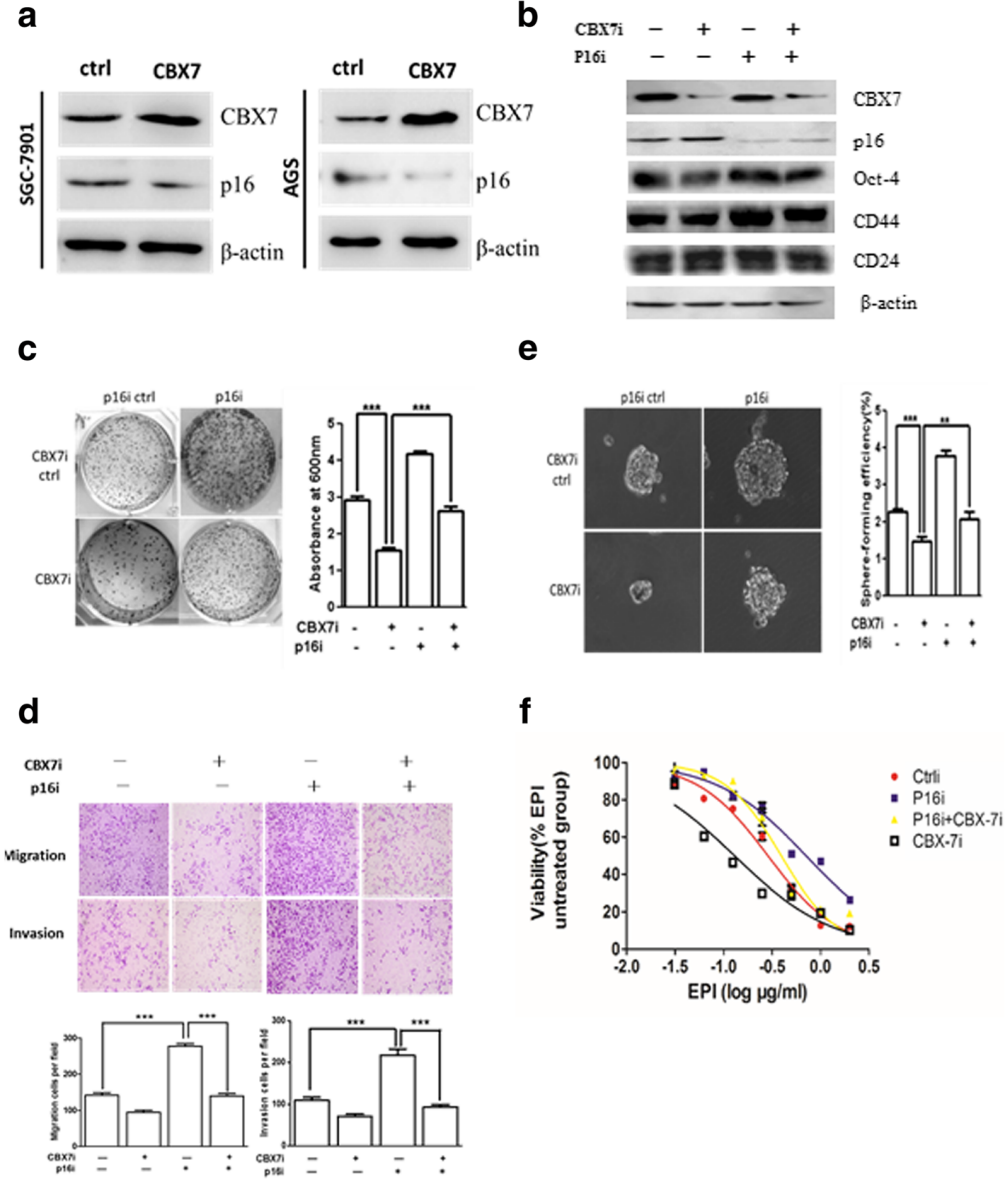
CBX7 regulates stem cell-like characteristics of gastric cancer cells through the inhibition of p16 (aka p16INK4a). **a** Stable overexpression of CBX7 downregulates the expression of p16 in GC cells. Western blot analysis was used to determine the levels of CBX7, p16, and β-actin in control and CBX7-overexpressing SGC-7901 and AGS cells. β-actin was used as an internal control. **b** Depletion of CBX7 upregulates p16. Western blot analysis was employed to detect CBX7 and p16 expression in SGC-7901 cells after transfection with p16 shRNA, CBX7 shRNA, or control construct. β-actin was used as an internal control. **c**–**f** Knockdown of p16 overcomes CBX7-induced GCSC phenotypes, as determined by colony formation (**c**), spheroid formation (**d**), migration and invasion (**e**), and chemoresistance assays (**f**). SGC-7901 cells were used to examine GCSC properties as described. EPI epirubicin. Error bars in all panels represent the mean ± SD (*P < 0.05, **P < 0.01 and ***P < 0.001, compared with the control)

It has been reported that CBX7 functions as an oncogene mainly through the downregulation of *p16INK4a/ARF* locus [33]. Hence, we determined whether CBX7 regulated the stem cell-like characteristics via the downregulation of p16. We overexpressed CBX7 in p16-expressing SGC-7901 and AGS gastric cancer cell lines and analyzed the expression of p16. Our results suggested that p16 expression was significantly decreased in both cell lines following CBX7 overexpression (Fig. 2a). Next, we employed a CBX7-targeting shRNA to deplete CBX7 expression in SGC-7901 cells and determined its effect on p16 expression, cell proliferation, sphere formation efficiency, chemosensitivity, migration, and invasion (Fig. 2b–f). As shown in Fig. 2b, interference of CBX7 (CBX7i) significantly reduced the expression of CBX7 and upregulated p16. Silencing CBX7 downregulated Oct-4, while co-expression of p16i could partially overcome the inhibitory effect of CBX7 silencing on Oct-4. To further determine the role of p16 in CBX7-medaited oncogenesis, we co-transfected a p16-targeting shRNA (p16i) in gastric cancer cells. The results revealed that interference of CBX7 resulted in reduced cell proliferation, sphere formation, migration, and invasion, whereas p16 downregulation resulted in upregulation of these oncogenic characteristics (Fig. 2c–e). Furthermore, co-transfection of CBX7- and p16-targeting shRNAs restored the proliferation, sphere formation, migration, and invasion of GC cells (Fig. 2c–e). Similarly, CBX7 downregulation increased chemosensitivity to epirubicin (EPI), whereas p16 shRNA restored chemoresistance in gastric cancer cells (Fig. 2f). Taken together, these results conceivably implicate that CBX7 facilitates cancer stem cell characteristics of gastric cancer cells via the downregulation of p16.

### AKT pathway contributes to CBX7-mediated cancer stem cell characteristics of gastric cancer cells

**Fig. 3:**
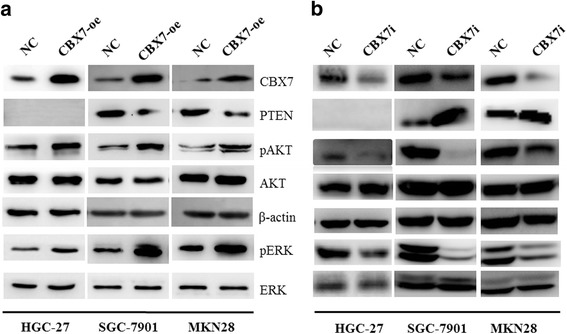
CBX7 induces AKT activation in GC cells. Expression of CBX7, pAKT, total AKT, PTEN, pERK, and total ERK in control (NC) and CBX7-overexpressing cells (**a**) and control (NC) and CBX7 knockdown cells (**b**) were determined using Western blot analysis. CBX7-overexpressing and CBX7-interfering cells were derived from HCG27, MKN28, and SGC-7901 cell lines as indicated

PcG proteins such as BMI1 are known to regulate oncogenesis via p16-dependent and independent pathways [25, 32, 34]. To determine whether CBX7 may regulate GCSC phenotype via p16-independent mechanisms, we analyzed the effect of CBX7 overexpression in MKN28, a p16-negative gastric cancer cell line. Our data suggested that CBX7 was significantly overexpressed in the oncospheres of p16-deleted MKN28 gastric cancer cells, and CBX7 overexpression resulted in the upregulation of GCSC phenotype such as sphere formation (Additional file 1: Figure S1). Because PcG protein BMI1 may regulate AKT and ERK pathways [32], we analyzed the expression of total AKT, phospho-AKT(p-AKT), total ERK and phospho-ERK (p-ERK) in control, CBX7-overexpressing (CBX7-oe), and CBX7-shRNA (CBX7i) gastric cancer cell lines. We also analyzed the expression of PTEN tumor suppressor, which is a major upstream regulator of AKT pathway. Our results indicated that CBX7 overexpression facilitated the activation of AKT and ERK, while depletion of CBX7 downregulated the levels of p-AKT and p-ERK in gastric cancer cells (Fig. 3a, b). Notably, we revealed that CBX7 negatively regulated PTEN expression in GC cells (Fig. 3a, b).

**Fig. 4:**
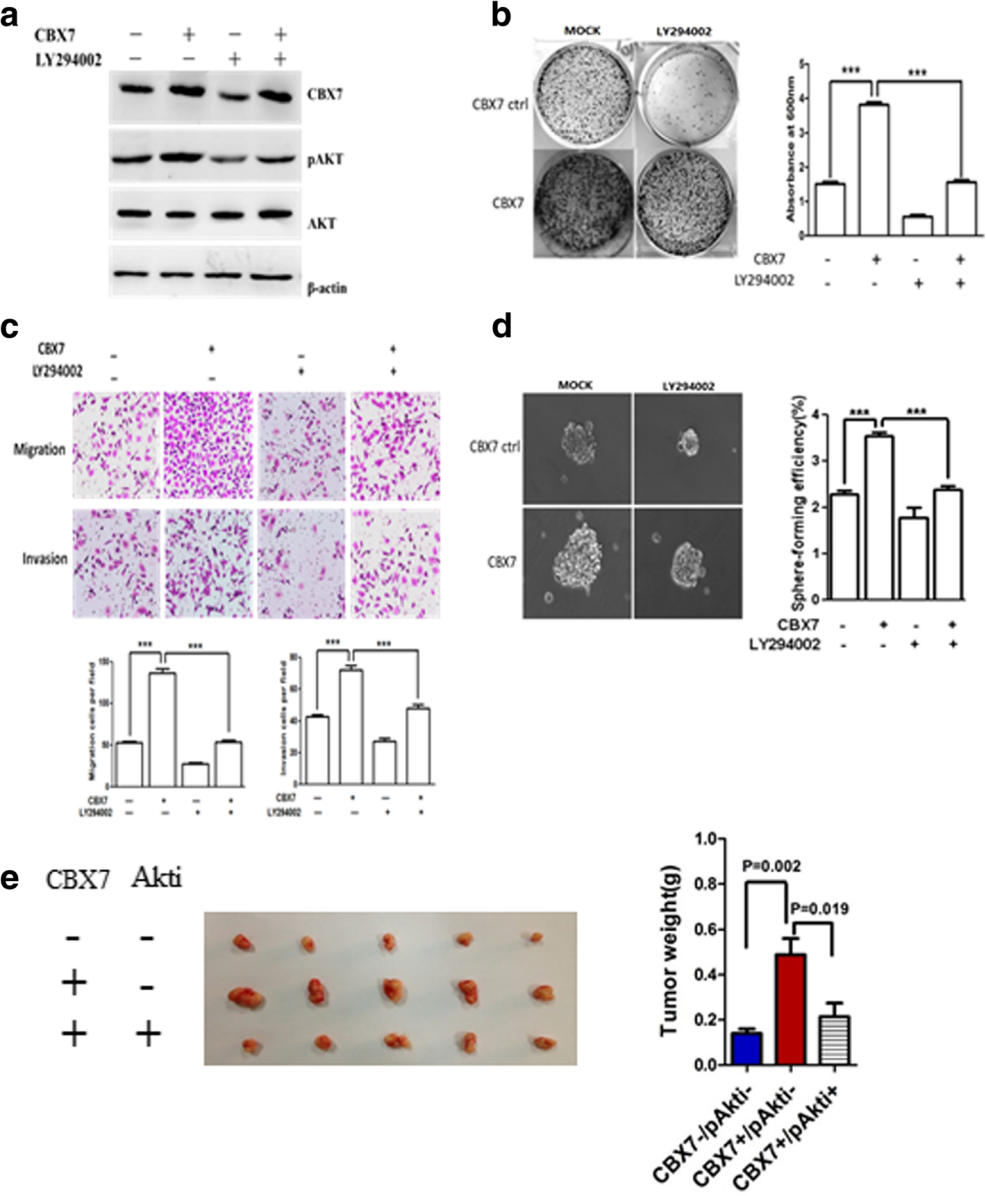
CBX7 regulates stem cell characteristics (GCSC phenotypes) of gastric cancer cells by activating AKT pathway. **a** Control and CBX7-overexpressing SGC-7901 cells were mock-treated or treated with LY294002 (50 μM) for 24 h. Western blot analysis was used to determine the expression of pAKT, AKT, CBX7, and β-actin. **b**–**d** Control and CBX7-overexpressing SGC-7901 cells were subjected to colony formation, migration, invasion, and spheroid colony formation assays in the absence or presence of LY294002. ***P < 0.001. **e** AKT knockdown suppresses CBX7-enhanced in vivo tumorigenicity in MGC-803 cells. In vivo tumorigenicity in CBX7, Akti, and CBX7+Akti cells was determined by subcutaneous injection of indicated gastric cancer cells into SCID mice

To further analyze the involvement of PI3K/AKT signaling pathway in GCSCs, control and CBX7-overexpressing gastric cancer cells were treated with LY294002, a specific inhibitor of PI3K/AKT pathway. Western blot analysis showed that LY294002 treatment could abrogate CBX7-mediated upregulation of p-AKT (Fig. 4a). Treatment with LY294002 also remarkably inhibited CBX7-mediated proliferation, migration, and invasion and sphere formation of GC cells (Figs. 4b–d). These results indicate that AKT pathway may contribute to the tumor-promoting effects of CBX7 in gastric cancer cells. In vivo xenograft tumor experiments also revealed that inhibition of AKT using stably expressed AKT shRNA (AKTi) can reverse tumor growth of CBX7-overexpressing MCB-803 cells (Fig. 4e).

**Fig. 5:**
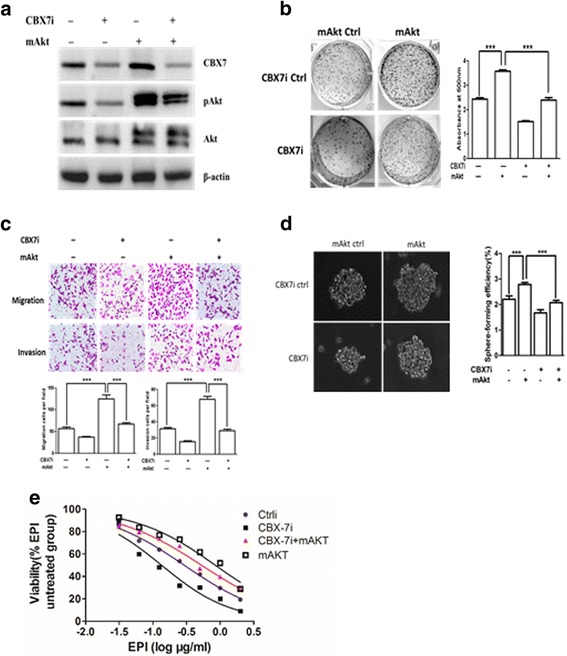
CBX7 regulates stem cell characteristics (GCSC phenotype) of gastric cancer cells by constitutive activation of AKT. **a** Western blot analysis was used to detect pAKT, total AKT, CBX7, and β-actin expression in control and SGC-7901 cells expressing pSRα-mAkt or CBX7i. β-actin served as an internal control. **b** Control and SGC-7901 cells expressing pSRα-mAkt and/or CBX7i were subjected to colony formation assay. **c** Control and SGC-7901 cells expressing pSRα-mAkt and/or CBX7i were subjected to migration and invasion assays. **d** Control and SGC-7901 cells expressing pSRα-mAkt and/or CBX7i were analyzed using spheroid formation assay. **e** The chemoresistance of control and SGC-7901 cells expressing pSRα-mAkt and/or CBX7i were determined. EPI epirubicin. ***P < 0.001

Next, we determined whether constitutive activation of AKT in gastric cancer cells can overcome the GCSC phenotype inhibiting effect of CBX7 shRNA. We overexpressed constitutively active form of AKT (pSRα-mAkt) in CBX7-depleted gastric cancer cells and determined its effect on oncogenic characteristics, including GCSC phenotypes (Fig. 5a). The results showed that the co-expression of myristoylated-akt (mAkt) could overcome the inhibitory effect of CBX7 interference on clonal proliferation, sphere formation, migration, invasion, and chemoresistance in gastric cancer cells (Fig. 5b–e). Thus, our data strongly suggest that CBX7 regulates stem cell-like properties of gastric cancer cells through AKT pathway.

### CBX7 regulates the expression of miR-21 through activating AKT-NF-κB pathway

**Fig. 6:**
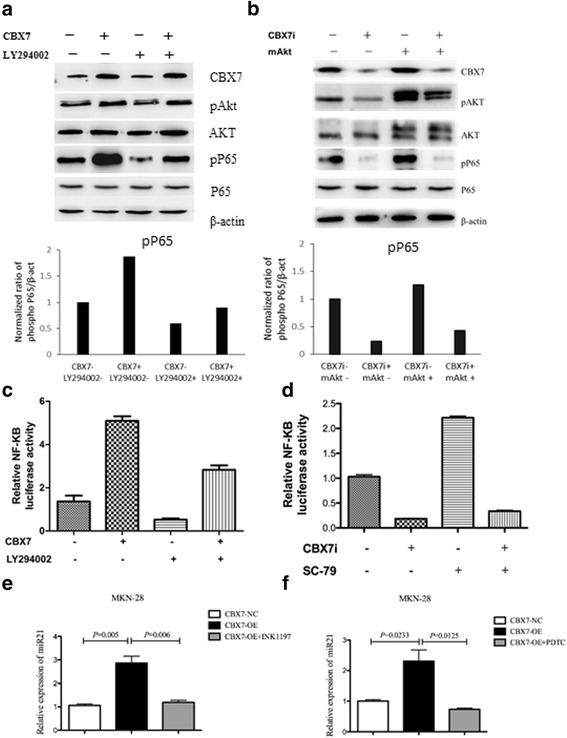
Activation of AKT by CBX7 leads to upregulation of NF-κB activity. **a** CBX7 overexpression upregulates the expression of phosphorylated P65 (pP65) and increases the expression of phosphorylated AKT (pAkt) in SGC7901 cells (upper panel) . The expression of CBX7, pAKT, total AKT (tAKT), pP65, and P65 was analyzed by Western blot analysis in gastric cancer cells. Treatment with AKT inhibitor LY294002 reduces the expression of pP65 induced by overexpression of mAKT. The lower panel represents normalized ratio of phospho P65/β-act of each group. **b** CBX7 knockdown downregulates the expression of pP65 and pAKT in SGC7901 cells (upper panel). The expression of CBX7, pAKT, total AKT (tAKT), pP65, and P65 was analyzed by Western blot analysis in gastric cancer cells. Constitutive active form of AKT can increase the expression of pP65 inhibited by CBX7 knockdown. The effect of CBX7 and AKT on pP65 and total P65 expression was determined using Western blot analysis. The lower panel represents normalized ratio of phospho P65/β-act of each group. **c** Treatment with AKT inhibitor LY294002 suppresses NF-κB transcriptional activity induced by CBX7 overexpression. Transcriptional activity of NF-κB in gastric cancer cells was determined by dual luciferase reporter assay. **d** AKT activator SC-79 abrogates CBX7 depletion-mediated suppression of NF-κB transcriptional activity. Transcriptional activity of NF-κB in gastric cancer cells was determined using dual luciferase reporter assay. **e** PI3K inhibitor INK1197 inhibits the expression of miR-21 induced by CBX7 overexpression. Fold change of miR-21 in gastric cancer cells was determined using quantitative RT-PCR analysis. **f** NF-κB inhibitor PDTC inhibits the expression of miR-21 induced by CBX7 overexpression. Fold change of miR-21 in gastric cancer cells was analyzed using quantitative RT-PCR analysis

Previously, we reported that BMI1, another member of the PcG family, could regulate stem cell-like properties of gastric cancer cells through the upregulation of miR-21 via AKT-NF-κB pathway [25]. Because our aforementioned data found that CBX7 activated AKT pathway in GC cells, we hypothesized that CBX7 may also upregulate miR-21 via AKT-NF-κB pathway. To validate this hypothesis, we determined whether CBX7 regulated NF-κB pathway through the activation of AKT. Using Western blot analysis, we found that indeed CBX7 upregulated cellular level of p-p65 in GC cells. Moreover, treatment with 50 μM LY294002 partially abrogated the upregulation of p-p65 following CBX7 expression (Fig. 6a, left panel). Furthermore, transfection of pSRα-mAkt partially restored the expression of p-p65 in CBX7-depleted GC cells (Fig. 6a, right panel). To further confirm these results, dual luciferase reporter assay was employed to detect NF-κB transcriptional activity following exogenous expression of CBX7. As shown in Fig. 6b, overexpression of CBX7 in SGC7901 cells significantly promoted the transcriptional activity of NF-κB, while treatment with PI3K/AKT inhibitor LY294002 abrogated CBX7-mediated upregulation of NF-κB transcriptional activity (Fig. 6b). In agreement with these findings, interference of CBX7 led to the impairment of NF-κB transcriptional activity, while the PI3K/Akt-specific activator SC-79 failed to restore decreased transcriptional activity of NF-κB caused by CBX7 depletion (Fig. 6c). In summary, these results strongly support the hypothesis that CBX7 regulates the transcriptional activity of NF-κB in gastric cancer cells by modulating AKT activation. Next, we determined whether CBX7 upregulated miR-21 through activating AKT-NF-κB pathway. As shown in Fig. 6d, e, CBX7 overexpression led to the upregulation of miR-21 in gastric cancer cells, while incubation with PI3K-specific inhibitor INK1197 abrogated CBX7-mediated expression of miR-21. These results suggest that CBX7 regulates miR-21 expression via AKT-NF-κB pathway.

### CBX7 regulates stem cell characteristics of gastric cancer cells via the upregulation of miR-21

**Fig. 7:**
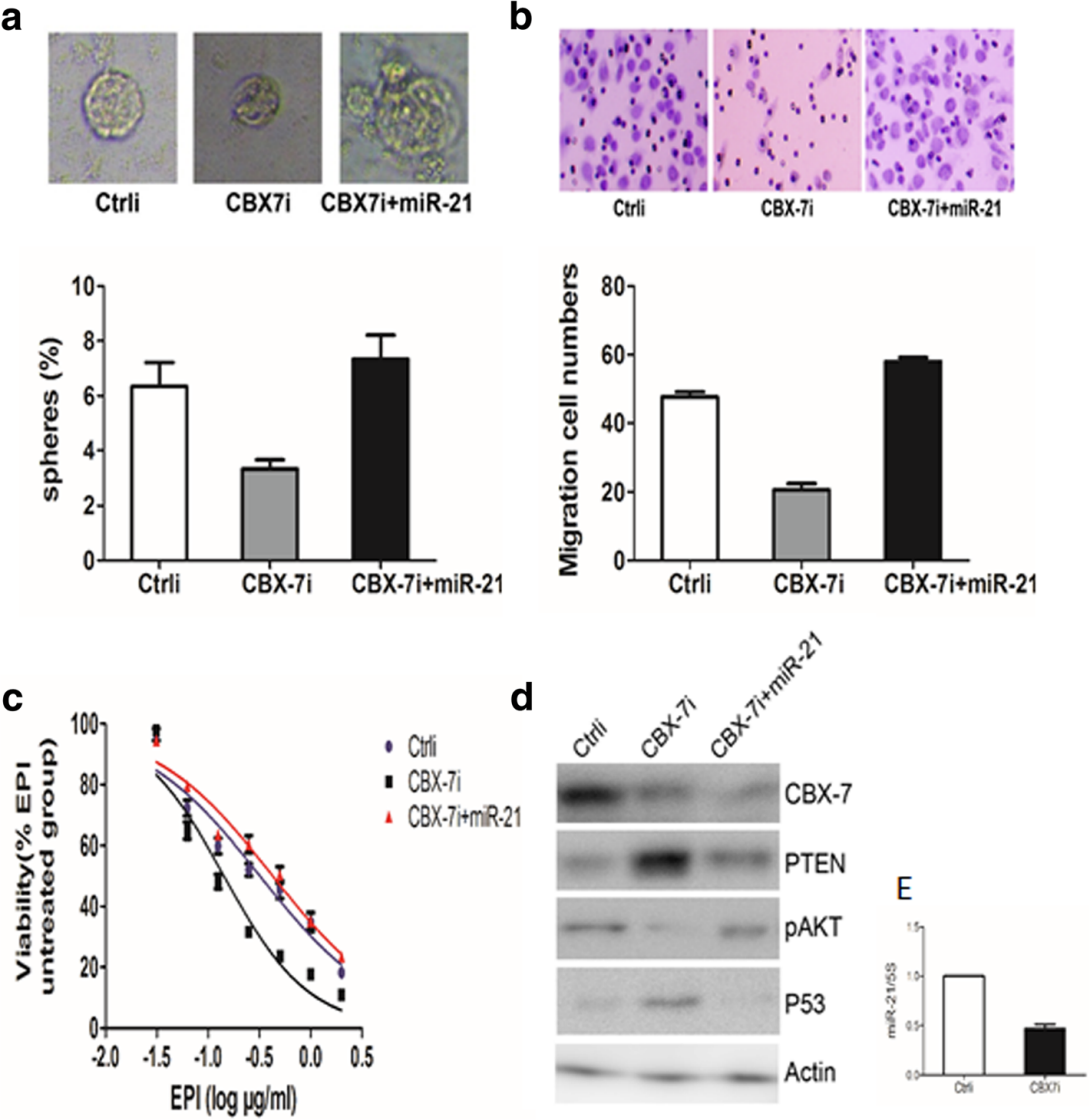
MiR-21 overexpression restores stem cell-like characteristics (GCSC phenotypes) of gastric cancer cells following CBX7 knockdown. **a** MiR-21 overexpression restores sphere formation of CBX7-depleted gastric cancer cells. Sphere formation in control (Ctrli), CBX7 knockdown (CBX7i), and CBX7i+miR-21 cells was analyzed using oncosphere-initiating medium. **b** MiR-21 overexpression enhances the migration of CBX7-depleted GC cells. The migratory capacities of Ctrli, CBX7i, and CBX7i+miR-21cells were examined by Transwell migration assay. **c** MiR-21 overexpression increases the chemoresistance of CBX7-depleted GC cells. Cell viability of Ctrli, CBX7i, and CBX7i+miR-21 cells treated was determined with CCK-8 assay following with exposure to different doses of EPI for 48 h. EPI epirubicin. **d** MiR-21 overexpression restores the expression of miR-21 target genes (PTEN and P53) in CBX7-depleted cells. The expression of CBX7 and miR-21 target genes (PTEN-AKT, P53) in Ctrli, CBX7i, and CBX7i+miR-21 cells was determined using Western blot analysis. **e** MiR-21 suppression in CBX7 knockdown cells was confirmed by qRT-PCR. Fold change of miR-21 in Ctrli, CBX7i cells was determined by qRT-PCR assay. Error bars in all panels represent the mean ± SD, *P < 0.05, **P < 0.01

As described above, our results suggest that CBX7 regulates miR-21 expression via AKT and NF-κB pathways. Hence, we speculated that CBX7 might regulate stem cell-like properties of gastric cancer cells via miR-21. As shown in Fig. 7a–c, overexpression of miR-21 could restore stem cell-like properties of CBX7-depleted GC cells, including cell self-renewal, cell migration, and chemoresistance. Intriguingly, some previous reports indicated that miR-21 posttrancriptionally downregulated the expression of PTEN to drive the activation of AKT pathway [35, 36]. We therefore speculated that altered expression of miR-21 might play a role in CBX7-triggered AKT pathway. In agreement with this hypothesis, overexpression of miR-21 partially rescued PTEN-AKT pathway and its downstream p53 signaling (Fig. 7d). Collectively, these results suggest that CBX7 regulate stem cell-like characteristics of gastric cancer cells via AKT-NF-κB pathway and miR-21.

## Discussion

Cancer stem cells (CSCs) belong to a class of functionally distinct subtype of cells in tumor cell population, with biological characteristics of self-renewal, multi-directional differentiation, high tumorigenicity, and resistance to radiotherapy and chemotherapy. These cells have been linked to tumor metastasis and disease recurrence. CSCs may form oncospheres in serum-free cultures supplemented with B27 and N2 supplements, as well as EGF and bFGF, making this technology widely used in the characterization of tumor cells with stem cell characteristics [4, 37]. Gastric CSCs have been characterized from gastric cancer tissues and gastric cancer cell lines using this method by Han [38] and Song et al. [39]. Cancer stem cells and normal stem cells have many common characteristics such as self-renewal and multi-directional differentiation [40]. Stem cell markers CD133, CD44, ABCG2, Oct4, Lgr5, and CD24 are also highly expressed in cancer stem cells. Using CD44 as a CSC marker protein, six GCSC lines were isolated by Takaishi et al. [7]. It was also reported that MKN-45, MKN-74, and NCI-N87 gastric cancer cell lines contain a considerable number of CD44-positive cells. These cells form spheres in a high efficiency and lead to tumor formation when injected in to the gastric cavity and skin of the SCID (severe combined immunodeficiency) mice, indicating that CD44-positive gastric cancer cell subsets have potent in vivo tumor-initiating capacity. Zhang et al. [9] reported that gastric cancer cells with positive expression of CD24 and CD44 exhibited stem cell-like properties, including self-renewal, multi-directional differentiation, and high tumorigenicity. Song et al. [39] confirmed that, compared to adherent cells, stem-like sphere-forming cells isolated from HGC-27, MGC-803, and MKN-45 gastric cancer cell lines expressed significantly higher levels of CD44, CD24, and CD133 proteins.

In the present work and a previous study, we successfully isolated GCSC spheres from gastric cancer cell lines, MKN28 and SGC-7901 [11, 25], and found that GCSCs express high levels of stem cell surface markers, including CD24, CD44, and OCT4. In addition, we found that GCSC spheres overexpress CBX7, suggesting that CBX7 may also participate in the regulation of GCSC characteristics. The function of CBX7 in malignant cancers is not clear and may depend on cellular context [19–23]. High expression of CBX7 was observed in tumors of blood system and prostate cancer, suggesting an oncogenic role. However, in some other cancers, lower expression of CBX7 was observed. Previously, it was reported that CBX7 was highly expressed in gastric tumors and that it may have an oncogenic role similar to other PcG proteins [23].

In the current study, we revealed that high expression of CBX7 in gastric carcinoma was positively correlated with lymph node metastasis and clinical stage. Functional studies showed that CBX7 could promote the growth of gastric cancer cells and colony formation, suggesting an oncogenic role in gastric carcinoma. In this regard, our experiments showed that overexpression of CBX7 promoted the formation of CSC-like spheres and facilitated the growth of in vivo subcutaneous transplanted tumor, cell invasion and migration, resistance to chemotherapy, and expression of stem cell markers, while downregulation of CBX7 could inhibit these oncogenic characteristics. Moreover, there was a positive correlation between the expression of CBX7 and stem cell markers OCT4 and CD133 in gastric carcinoma tissues. These results suggested that CBX7 positively regulates cancer stem cell characteristics of gastric cancer. Next, we investigated the potential mechanisms by which CBX7 regulates stem cell-like characteristics of gastric cancer cells. A previous study showed that CBX7 might inhibit the expression of p16 to prolong cellular life span [41], promote oncogenic phenotypes in lymphoma [33], and control the growth of normal and tumor-derived prostate cells [27]. BMI1-mediated suppression of p16INK4a is also known to regulate stem cell phenotypes [42]. Therefore, it is possible that CBX7 also regulates stem cell-like characteristics of gastric cancer via the repression of p16. Indeed, our studies strongly suggest that CBX7 regulates expression of p16 in p16-expressing gastric cancer cell lines SGC-7901 and AGS. Further functional reconstitution experiments found that p16 knockdown could partially restore the decreased cell proliferation, serum-free sphere formation, resistance to chemotherapy, and invasion and migration of cells caused by CBX7 gene silencing. Intriguingly, some reports suggested a direct involvement of p16 in the regulation of NF-κB pathway [40, 43, 44] These findings highlight a potential interplay between CBX7-mediated repression of p16 and activation of AKT-NF-κB signaling. Indeed, we found that overexpression of mAKT could only partially restore the expression of p-p65 following CBX7 depletion in GC cells, suggesting a potential role of p16 in the repression of NF-κB pathway (Fig. 6a, c). These findings imply a complicated role of p16 in CBX7-intiated gastric cancer progression. In summary, our experimental results showed that CBX7 could regulate gastric cancer stem cell phenotype by inhibiting p16INK4a.

Given the facts that p16 knockdown can only partially restore GCSC characteristics in CBX7-deficient gastric cancer cells and that CBX7 can also regulate GCSC phenotype in p16-defcient MKN-28 gastric cancer cells, we speculate that CBX7 may regulate gastric cancer stem cell properties through pathways that are independent of p16. AKT and ERK signaling pathways are known to regulate cell proliferation and apoptosis, epithelial mesenchymal transition (EMT), tumor angiogenesis and metastasis, and resistance of chemotherapy through a series of downstream molecules [45–48]. AKT is also a downstream effector of BMI1, which is a member of the PcG family and a well-known regulator of CSC phenotype. A study by Guo et al. found that BMI1 could promote breast cancer cell proliferation through the activation of AKT [32]. Because both BMI1 and CBX7 are essential constituents of the PRC1, we speculated that CBX7 might function through AKT and EKR pathways. In this study, we found that overexpression of CBX7 in gastric carcinoma cells led to increased expression of phosphorylated AKT (pAKT) and phosphorylated EKR (pERK), while knockdown of CBX7 decreased the levels of pERK and pAKT, suggesting that CBX7 can regulate AKT and EKR pathways. Furthermore, expression of CBX7 positively correlates with the expression of pAKT in gastric carcinoma but not with the expression of pERK, suggesting that AKT may be an important downstream effector of CBX7 similar to BMI1. Hence, we further studied the role of AKT as a downstream effector of CBX7 and found that treatment with PI3K/Akt inhibitor LY294002 could partially inhibit enhanced gastric cancer cell proliferation, migration and invasion, and ability of sphere formation induced by high expression of CBX7, while exogenous high expression of constitutively active AKT (mAKT) can reverse decreased gastric cancer cell proliferation, migration and invasion, and ability of sphere formation due to CBX7 interference. These experimental results validated the hypothesis that CBX7 could regulate proliferation and stem cell-like properties of gastric cancer cells through the activation of Akt pathway. Further biochemical analyses suggested that CBX7 could activate AKT pathway via the repression of PTEN as reported for BMI1 [49]. However, CBX7 does not appear to directly bind to PTEN promoter in gastric cancer cells (data not shown).

We previously reported that BMI1 could regulate stem cell properties of gastric cancer through the upregulation of miR-21 by activating AKT-NF-κB pathway. Because CBX7 can also activate AKT, we speculated that CBX7 might also upregulate miR-21 by regulating AKT-NF-κB pathway and that CBX7-intiated AKT-NF-κB-miR-21 pathway might contribute to GCSC phenotype. Indeed, we found that PI3K/Akt-specific inhibitor LY294002 could partially inhibit the upregulation of pP65 and the enhanced transcriptional activity of NF-κB caused by the upregulation of CBX7, while exogenous expression of pSRα-mAkt can partially restore the inhibition of pP65 by the downregulation of CBX7. Furthermore, PI3K/Akt-specific activator SC-79 can restore the transcriptional activity of NF-κB caused by the downregulation of CBX7. Along these lines, we also found that PI3K inhibitor INK1197 and NF-κB inhibitor PDTC treatment can inhibit the increase of miR-21 expression induced by CBX7 overexpression (Fig. 3e, f). These results suggested that CBX7 regulated miR-21 expression via AKT-NF-κB pathway, which in turn could possibly regulate stem cell-like properties of gastric cancer cells. Indeed, exogenous miR-21 overexpression can reverse the inhibition of stem cell-like properties (cell self-renewal, cell migration and resistance to chemotherapy) caused by CBX7 interference. These results suggested that CBX7 might regulate the stem cell-like characteristics of gastric cancer cells through miR-21. In summary, we propose that CBX7-AKT-NF-κB-miR-21 pathway is an important determinant of GCSC phenotype and that targeting this pathway may provide new therapeutic opportunities for gastric cancer treatment.

## Conclusion

In conclusion, our data indicate that CBX7 could potentiate stem cell-like characteristics of GC cells through repressing p16 and activating AKT-NF-κB-miR-21 pathway. Restoring the expression of p16 partially abrogated CBX7-triggered stem cell properties of gastric cancer cells. Moreover, we also uncovered AKT-NF-κB-miR-21 signaling axis as a critical downstream effector of CBX7-initiated stem cell characteristics. These findings provide an insightful view into the mechanisms underlying GC stem cell initiation and expansion.

## Additional files

Additional file 1: Figure S1. CBX7 positively regulates stem cell-like properties of gastric cancer cells in MKN28 cell line. (A) Tumorigenic spheres are derived from MKN28 gastric cancer cell line in serum-free media containing EGF and bFGF. (B) Tumorigenic spheres overexpressed CBX7 and stem cell markers including Oct-4, CD44, and CD24. The expression of these proteins was analyzed by Western blot. (C) Expression levels of CBX7 protein were detected by Western Blot in MKN28 cells expressing control vector or CBX7. β-actin was used as an internal control. (D) The colony-forming capability of the CBX7 stably expressing cells detected by seeding 1000 cells and an incubation time ranging from 10 days. The colonies fixed and crystal violet-stained. To solubilize the crystal violet, 200 μl of 10% acetate was added to each well and mixed. A 100 μl aliquot was removed to a new well, and the absorbance was read at 600 nm. (E) The migration and invasion assays using the Corning chamber were used to test the CBX7 stably expressing cell lines, compared with the control. (F) Representative images of CCSC spheres after transfected empty vector and CBX7 plasmid respectively. CBX7 expression enhance spheroid-forming capability inMKN28 cells. (TIFF 1115 kb)

Additional file 2: Figure S2. Downregulation of CBX7 reduce stem cell-like properties of gastric cancer cells in SCG7901 cell line. (A) Expression levels of CBX7 protein were detected by Western Blot in SCG7901 cells expressing control vector or CBX7i. β-actin was used as an internal control. (B) The colony-forming capability after downregulation of CBX7 detected by seeding 1000 cells and an incubation time ranging from 10 days. The colonies fixed and crystal violet-stained. (C) Representative images of CCSC spheres after downregulation of CBX7. CBX7 downregulation reduces spheroid-forming capability in SGC-7901 cells. (D) The migration and invasion assays using the Corning chamber were used to test the CBX7 inhibition cell lines, compared with the control. (TIFF 1192 kb)

Additional file 3: Figure S3. Downregulation of CBX7 reduce stem cell-like properties of gastric cancer cells in MKN28 cell line. (A) Expression levels of CBX7 protein were detected by Western Blot in SCG7901 cells expressing control vector or CBX7i. β-actin was used as an internal control. (B) The colony-forming capability after downregulation of CBX7 detected by seeding 1000 cells and an incubation time ranging from 10 days. The colonies fixed and crystal violet-stained. (C) Representative images of CCSC spheres after downregulation of CBX7. CBX7 downregulation reduces spheroid-forming capability in MKN28 cells. (TIFF 469 kb)

## Abbreviations

CBX7: Chromobox protein homolog 7
CCK8: Cell Counting Kit-8
CSC: Cancer stem cell
EMT: Epithelial-mesenchymal transition
EPI: Epirubicin
GC: Gastric cancer
GCSCs: Gastric cancer stem cells
IHC: Immunohistochemical analysis
miRNA: microRNA
PcG: Polycomb group
PRCs: Polycomb repressive complexes
QRT-PCR: Quantitative real-time PCR
SCF: Spheroid colony formation
SCID: Severe combined immunodeficiency
