# Supplementary figures and images for: CBX7 regulates stem cell-like properties of gastric cancer cells via p16 and AKT-NF-κB-miR-21 pathways
Source: J Hematol Oncol. 2018 Feb 8;11:17. doi: 10.1186/s13045-018-0562-z (PMC5806263; doi:10.1186/s13045-018-0562-z)

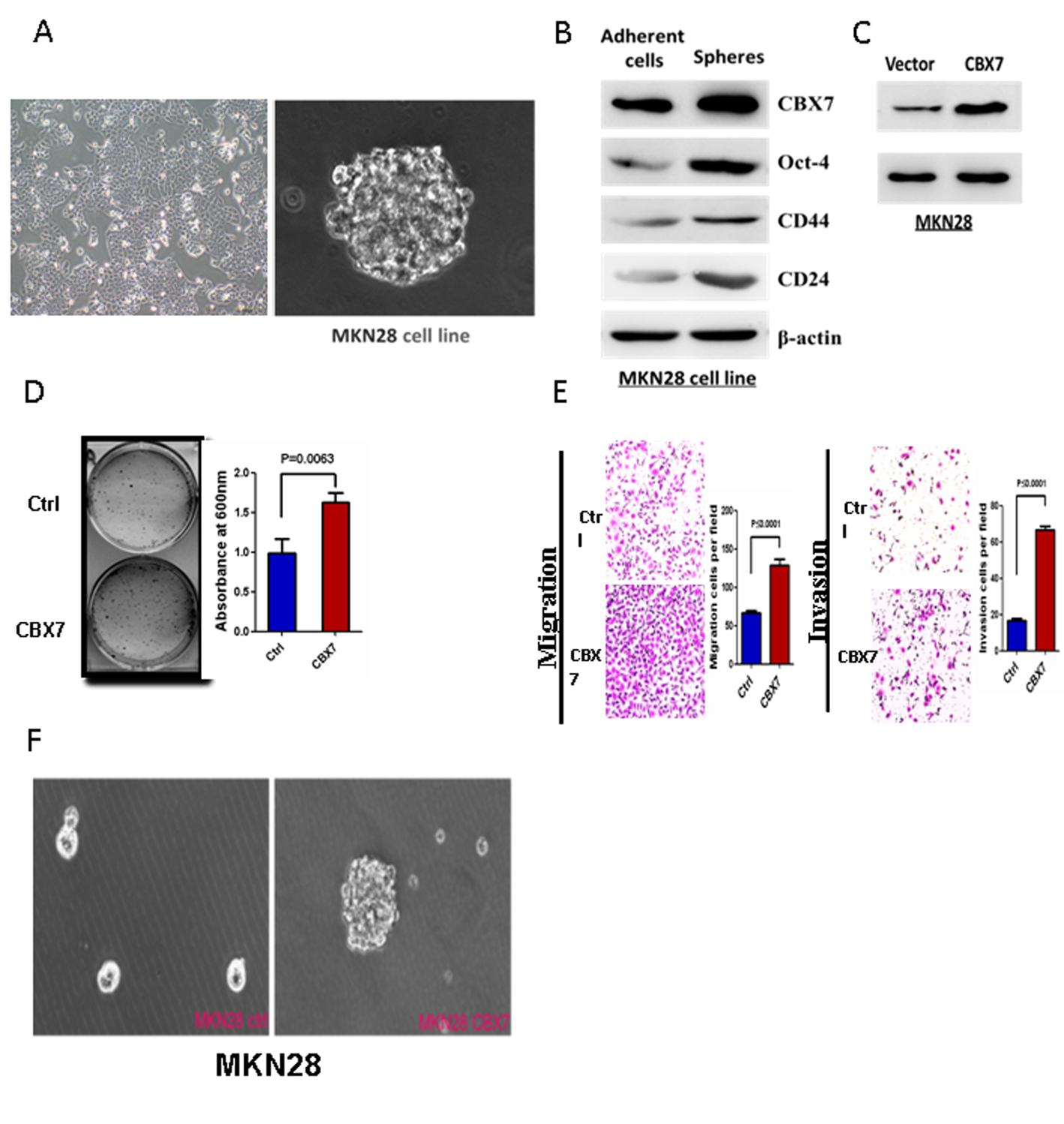

Supplement: Supplementary file 1 — CBX7 positively regulates stem cell-like properties of gastric cancer cells in MKN28 cell line. (A) Tumorigenic spheres are derived from MKN28 gastric cancer cell line in serum-free media containing EGF and bFGF. (B) Tumorigenic spheres overexpressed CBX7 and stem cell markers including Oct-4, CD44, and CD24. The expression of these proteins was analyzed by Western blot. (C) Expression levels of CBX7 protein were detected by Western Blot in MKN28 cells expressing control vector or CBX7. β-actin was used as an internal control. (D) The colony-forming capability of the CBX7 stably expressing cells detected by seeding 1000 cells and an incubation time ranging from 10 days. The colonies fixed and crystal violet-stained. To solubilize the crystal violet, 200 μl of 10% acetate was added to each well and mixed. A 100 μl aliquot was removed to a new well, and the absorbance was read at 600 nm. (E) The migration and invasion assays using the Corning chamber were used to test the CBX7 stably expressing cell lines, compared with the control. (F) Representative images of CCSC spheres after transfected empty vector and CBX7 plasmid respectively. CBX7 expression enhance spheroid-forming capability inMKN28 cells. (TIFF 1115 kb) [file 13045_2018_562_MOESM1_ESM.tif]

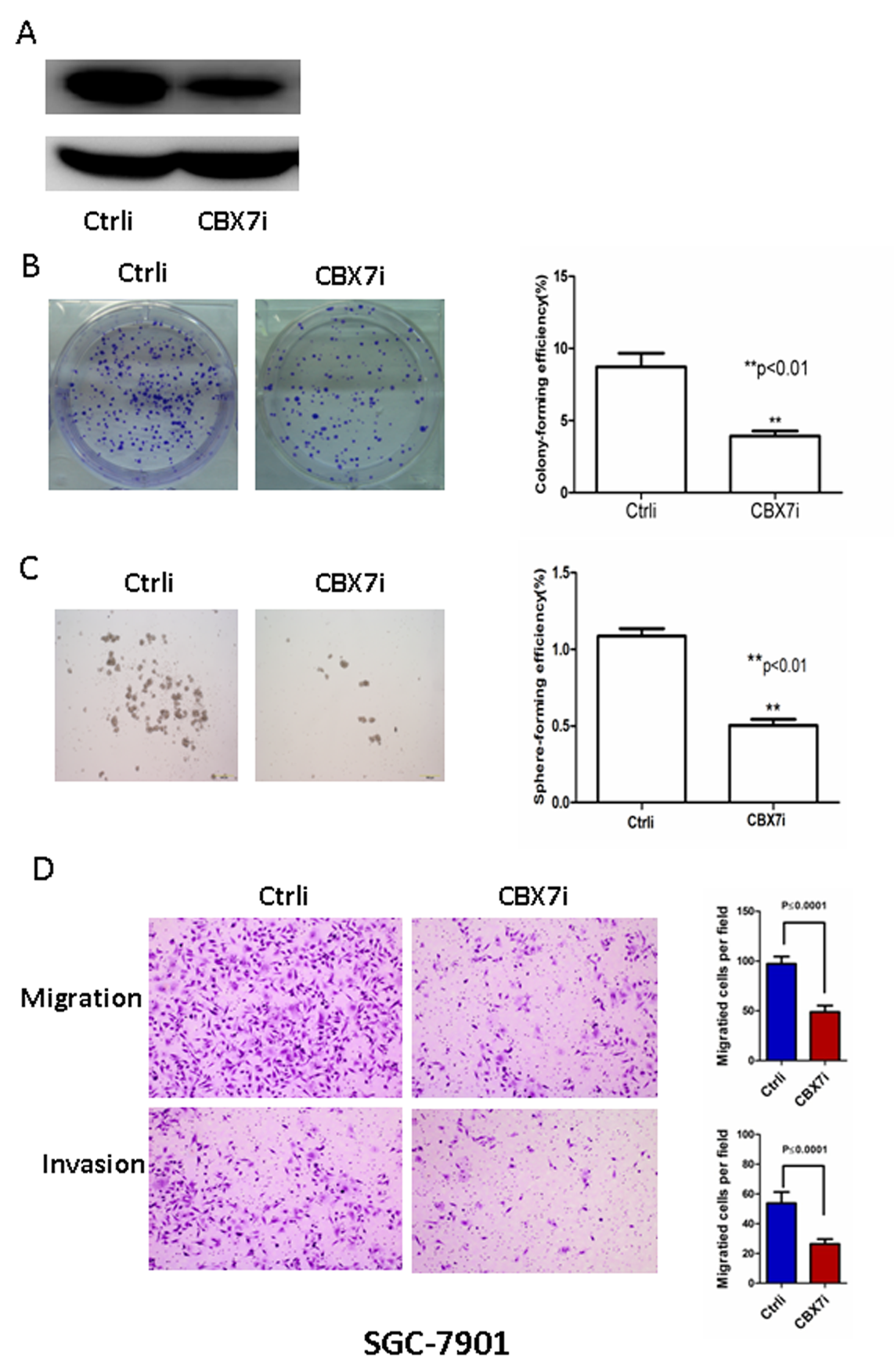

Supplement: Supplementary file 2 — Downregulation of CBX7 reduce stem cell-like properties of gastric cancer cells in SCG7901 cell line. (A) Expression levels of CBX7 protein were detected by Western Blot in SCG7901 cells expressing control vector or CBX7i. β-actin was used as an internal control. (B) The colony-forming capability after downregulation of CBX7 detected by seeding 1000 cells and an incubation time ranging from 10 days. The colonies fixed and crystal violet-stained. (C) Representative images of CCSC spheres after downregulation of CBX7. CBX7 downregulation reduces spheroid-forming capability in SGC-7901 cells. (D) The migration and invasion assays using the Corning chamber were used to test the CBX7 inhibition cell lines, compared with the control. (TIFF 1192 kb) [file 13045_2018_562_MOESM2_ESM.tif]

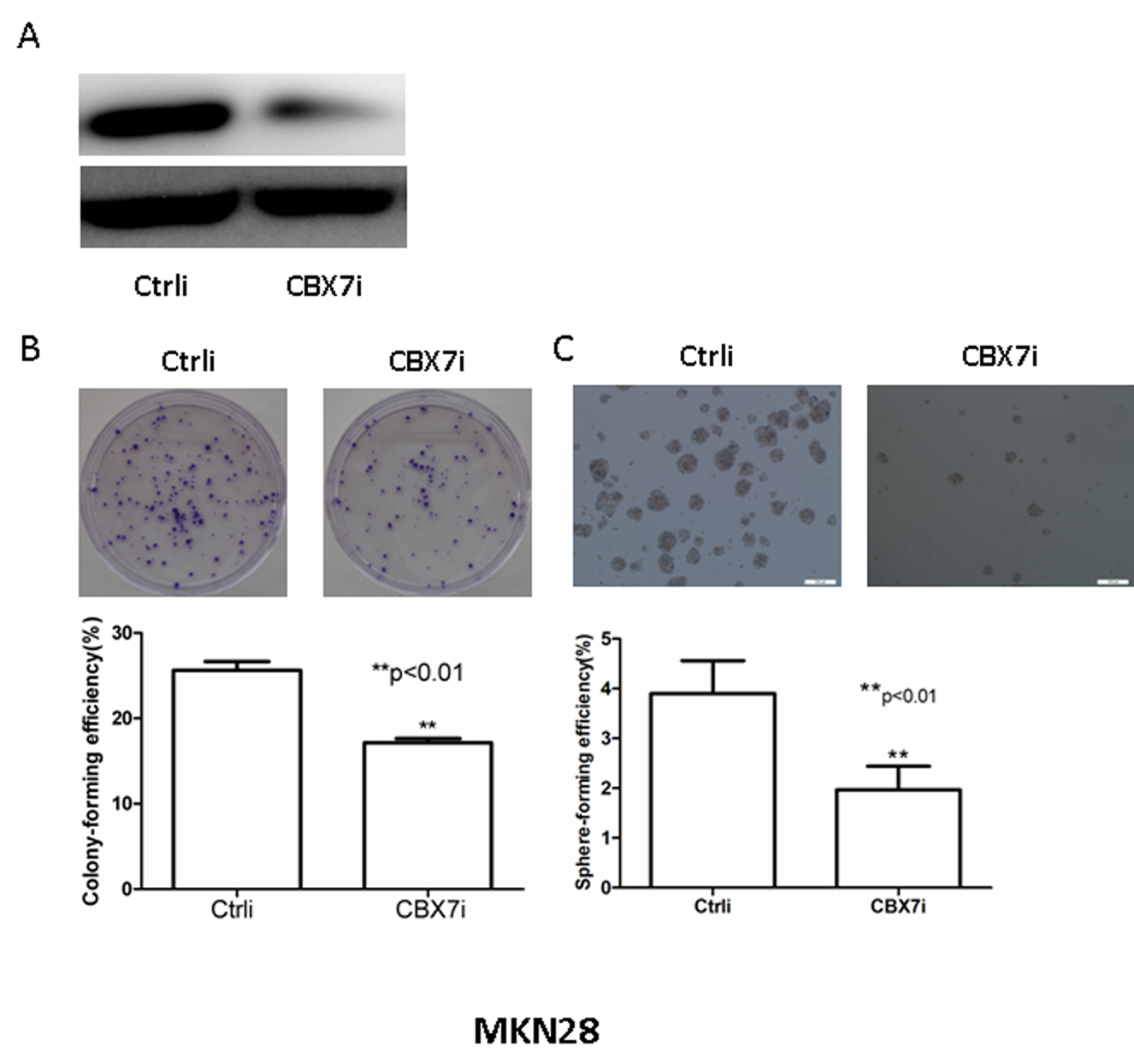

Supplement: Supplementary file 3 — Downregulation of CBX7 reduce stem cell-like properties of gastric cancer cells in MKN28 cell line. (A) Expression levels of CBX7 protein were detected by Western Blot in SCG7901 cells expressing control vector or CBX7i. β-actin was used as an internal control. (B) The colony-forming capability after downregulation of CBX7 detected by seeding 1000 cells and an incubation time ranging from 10 days. The colonies fixed and crystal violet-stained. (C) Representative images of CCSC spheres after downregulation of CBX7. CBX7 downregulation reduces spheroid-forming capability in MKN28 cells. (TIFF 469 kb) [file 13045_2018_562_MOESM3_ESM.tif]
